# Data related to dislocation density-based constitutive modeling of the tensile behavior of lath martensitic press hardening steel

**DOI:** 10.1016/j.dib.2017.09.034

**Published:** 2017-09-22

**Authors:** Kyoung-Rae Jo, Eun-Jung Seo, Dimas Hand Sulistiyo, Jin-Kyung Kim, Seong-Woo Kim, Bruno C. De Cooman

**Affiliations:** a Graduate Institute of Ferrous Technology, Pohang University of Science and Technology, Pohang 37673, Republic of Korea; b POSCO Technical Research Laboratories, Gwangyang 57807, Republic of Korea

## Abstract

The data presented in this article are related to the research article entitled “On the plasticity mechanisms of lath martensitic steel” (Jo et al., 2017) [1]. The strain hardening behavior during tensile deformation of a lath martensitic press hardening steel was described using a dislocation density-based constitutive model. The Kubin–Estrin model was used to describe strain hardening of the material from the evolution of coupled dislocation densities of mobile and immobile forest dislocation. The data presented provide insight into the complex deformation behavior of lath martensitic steel.

**Specifications Table**

**Table:** 

Subject area	*Materials Science*
More specific subject area	*Physical Metallurgy*
Type of data	*Table, Graphs*
How data was acquired	*Constitutive modeling*
Data format	*Raw and analyzed*
Experimental factors	*A cold-rolled 0.35 wt% C press hardening steel (PHS) was used. The tensile samples were austenitized and then quenched to room temperature in order to make fully martensitic microstructure.*
Experimental features	*The Kubin–Estrin model was used to describe the strain hardening behavior during tensile deformation of PHS from the evolution of the coupled densities of mobile dislocations, ρm, and immobile forest dislocations, ρf.*
Data source location	*Graduate Institute of Ferrous Technology, Pohang University of Science and Technology, Pohang, Korea*
Data accessibility	*The data are available with this article.*

**Value of the data**

• The data can be used to explain strain hardening behavior of lath martensitic steel.
• The data provide a foundation for more accurate modeling of strain hardening behavior of lath martensitic steel.
• The data may be compared with the tensile behavior of other lath martensitic steels.

## 1 Data

The strain hardening behavior during tensile deformation of a lath martensitic press hardening steel (PHS) was described using a dislocation density-based constitutive model. The Kubin–Estrin model was used to describe strain hardening of the material from the evolution of coupled dislocation densities of mobile and immobile forest dislocation. Two models with different parameter values are presented, and the results include stress–strain curves and the evolution of mobile and forest dislocation density with strain, calculated by the models. The parameter values used for modeling are presented in a table.

## 2 Experimental design, materials and methods

**Fig. 1:**
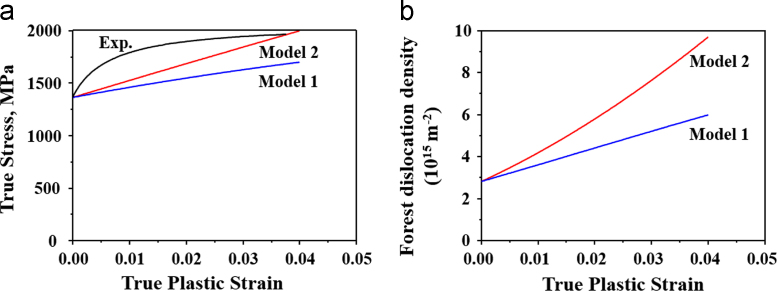
(a) Comparison of the experimental true stress-strain curve of the PHS and the calculated true stress–strain curve from model 1 and model 2. (b) The calculated strain dependence of forest dislocation density from model 1 and model 2.

A cold-rolled 0.35 wt% C PHS was used [1]. The tensile samples were austenitized and then quenched to room temperature in order to make fully martensitic microstructure. The specimens were tested in tension in an electromechanical universal testing machine using a strain rate of 10^−3^ s^−1^. The experimental true stress-strain curve of the as-quenched PHS is shown in Fig. 1.

The conventional yield strength (YS), i.e. 0.2% offset stress, of lath martensitic steel is generally high as compared to other steels. However, micro-yielding can occur at stresses lower than the 0.2% offset stress. Due to the absence of a clear yield point in the flow curve of lath martensitic steel, the 0.2% offset YS is considered as the YS of the material in the present work. The equation proposed by Galindo-Nava and Rivera-Diaz-del-Castillo was used to calculate the YS of the PHS [2]:

(1) $$\sigma_{\mathrm{Martensite}}\;=\;\sigma_{0}\;+\;\frac{300}{\sqrt{d_{\mathrm{block}}}}\;+\;0.25\mathrm{MGb}\sqrt{\rho }$$

Here, *σ*_0_ is contributions from Peierls stress and solid solution strengthening, *M* is the Taylor factor, *G* is the shear modulus, b is the magnitude of the Burgers vector and *ρ* is the total dislocation density. The equation for *σ*_0_ derived by Rodriguez and Gutierrez [3] yielded 201 MPa considering the chemical composition of the investigated PHS. The present work did not consider solid solution hardening by carbon. Using the block size of 500 nm, the second term in Eq. (1) was estimated to be 424 MPa. The average initial forest dislocation density in the PHS was estimated to be 2.21×10^15^ m^−2^ by subtracting the sum of contributions from the first term, i.e. 201 MPa, and the packet size strengthening term, i.e. 424 MPa, from the experimental YS, 1354 MPa. The estimated dislocation density is in a reasonable agreement with the measured dislocation density of a Fe-0.4 wt%C martensitic steel, i.e. 1.42×10^15^ m^−2^, reported by Morito et al. [4].

The Kubin–Estrin model was used to describe strain hardening behavior of the PHS from the evolution of coupled densities of mobile dislocations, *ρ*_m_, and immobile forest dislocations, *ρ*_f_ [5], [6].

(2) $$\frac{d\rho_{m}}{d\epsilon }\;=\;M\left\lceil \frac{C_{1}}{b^{2}}\left(\;\frac{\rho_{f}}{\rho_{m}}\right)\;-\;C_{2}\rho_{m}\;-\;\frac{C_{3}}{b}\sqrt{\rho_{f}}\right\rceil \frac{d\rho_{f}}{d\epsilon }\;=\;M[C_{2}\rho_{m}\;+\;\frac{C_{3}}{b}\sqrt{\rho_{f}}\;-\;C_{4}\rho_{f}]$$

In these equations, *C*_1_ specifies the magnitude of the dislocation generation term, with forest obstacles acting as pinning points for fixed dislocation sources. *C*_2_ takes into account the mobile dislocation density decrease by interactions between mobile dislocations. *C*_3_ describes the immobilization of mobile dislocations assuming a spatially organized forest structure. *C*_4_ is associated with dynamic recovery by rearrangement and annihilation of forest dislocations by climb or cross slip. *C*_2_ and *C*_4_ account for thermally activated mechanisms such as cross-slip and climb [5].

The parameters *C*_1_, *C*_2_, *C*_3_ and *C*_4_ used in the present work are listed in Table 1. The parameters in original Kubin–Estrin model were chosen based on typical FCC metals and alloys [6]. In the present work, a much higher values of *C*_3_ were used to describe the high initial work hardening of PHS as compared to the value in the original Kubin-Estrin model. The numerical values of the parameters were *G*=81.6 GPa, *b*=0.248 nm and *M*=3.067.

**Table 1:** Parameter values used for numerical simulations.

Parameters	Original Kubin–Estrin model	Model 1	Model 2
*C*_1_/*b*^2^	10^15^/3	10^15^/3	10^15^/3
*C*_2_	0.606	1.42	0.7
*C*_3_/b	10^8^/3.3	9 × 10^8^	9 × 10^8^
*C*_4_	3.33	7	3.5

Two models were analyzed. In the first model, i.e. model 1, high values of *C*_2_ and *C*_4_ were used since BCC metals and alloys generally have higher cross-slip activity as compared to FCC metals and alloys. As shown in Fig. 1(a), the experimental flow stress is much higher than the calculated flow stress by model 1. In the second model, i.e. model 2, lower values of *C*_2_ and *C*_4_ were used in order to match the experimental and calculated flow stresses. Neither model could however describe the high initial work hardening rate shown in the experimental flow curve.
