# Supplementary material for: Data related to dislocation density-based constitutive modeling of the tensile behavior of lath martensitic press hardening steel
Source: Data Brief. 2017 Sep 22;15:240–3. doi: 10.1016/j.dib.2017.09.034 (PMC5633252; doi:10.1016/j.dib.2017.09.034)
Supplement: Supplementary file 1 — Transparency document [file mmc1.docx]

**The authors declare that they have no conflict of interest.**
